# The atypical mammalian ligand Delta-like homologue 1 (Dlk1) can regulate Notch signalling in *Drosophila*

**DOI:** 10.1186/1471-213X-8-11

**Published:** 2008-01-31

**Authors:** Sarah J Bray, Shuji Takada, Emma Harrison, Shing-Chuan Shen, Anne C Ferguson-Smith

**Affiliations:** 1 Department of Physiology, Development, and Neuroscience, University of Cambridge, Downing Street, Cambridge, CB2 3DY, UK

## Abstract

**Background:**

Mammalian *Delta-like 1 *(*Dlk-1*) protein shares homology with Notch ligands but lacks a critical receptor-binding domain. Thus it is unclear whether it is able to interact with Notch *in vivo*. Unlike mammals, *Drosophila *have a single Notch receptor allowing a simple *in vivo *assay for mammalian *Dlk1 *function.

**Results:**

Here we show that membrane-bound DLK1 can regulate Notch leading to altered cellular distribution of Notch itself and inhibiting expression of Notch target genes. The resulting adult phenotypes are indicative of reduced Notch function and are enhanced by *Notch *mutations, confirming that DLK1 action is antagonistic. In addition, cells expressing an alternative *Dlk1 *isoform exhibit alterations in cell size, functions previously not attributed to Notch suggesting that DLK1 might also act via an alternative target.

**Conclusion:**

Our results demonstrate that DLK1 can regulate the Notch receptor despite its atypical structure.

## Background

**Figure 1:**
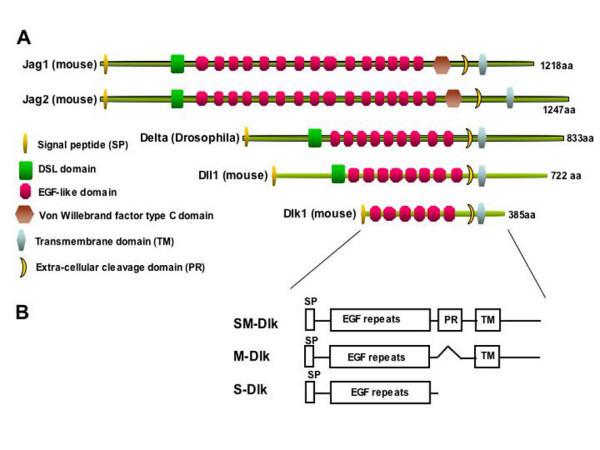
**Structure of Dlk and the 3 isoforms**. A) Schematic representation of several members of the Delta-Serrate family of Notch ligands. Comparative structure is shown including the EGF-repeats, signal peptide, extracellular proteolytic cleavage domain and transmembrane domain found in all the ligands in addition to the DSL domain that is missing in mouse *DLK1*. B) Schematic representation of the three DLK1 isoforms expressed in Drosophila. The M construct produces the membrane-bound isoform of DLK1, which lacks a proteolytic cleavage site. The SM construct produces the full-length DLK1 containing the proteolytic cleavage domain and the S form is engineered to produce the isoform normally generated by proteolytic cleavage.

The protein encoded by the mammalian *Delta-like 1 *(*Dlk1*) is related to members of the Notch-Delta family of signalling molecules, but differs in several key respects from other members of this family. *Dlk1 *produces multiple alternatively spliced transcripts, giving rise to protein isoforms which are either membrane-bound or proteolytically cleaved and secreted (Fig. 1; [1]). *Dlk1 *has evoked considerable interest because in mammals it is a paternally expressed imprinted gene that is epigenetically regulated, and defective imprinting of *Dlk1 *results in developmental abnormalities [2,3]. Furthermore, targeted deletion of *Dlk1 *in mice results in growth retardation, skeletal malformation and obesity [4] and transgenic mice ectopically expressing ovine *Dlk1 *in some muscle fibres, exhibit muscle fibre hypertrophy [5]. The developmental mechanisms leading to these abnormalities are unknown and in particular it is unclear the extent to which this protein has the capability of regulating Notch signalling *in vivo*, because it lacks an essential extracellular DSL domain common to all known Notch ligands.

Notch-ligands are transmembrane proteins that are characterised by a series of EGF-repeats in their extracellular domain, and an N-terminal domain, referred to as the DSL domain (Fig. 1; [6-8]). The latter appears to be critical for the interactions between the ligands and the Notch receptor [6,9]. Although DLK1 contains 6 EGF-repeats that are closely related to those found in Delta and Serrate/Jagged, and can bind to Notch EGF repeats in a yeast two-hybrid assay [10], it lacks the N-terminal DSL-domain which is required for receptor binding and activity of known ligands (Fig. 1); it also differs from characterised Notch ligands because its intracellular domain is considerably shorter. Truncation of the intracellular domain renders *Drosophila *or *Xenopus *Delta proteins incapable of activating the receptor [11-13]. Both its lack of a DSL-domain and its short intracellular domain suggest therefore that DLK1 would not be able to function as a Notch ligand. However, as an inverse correlation between *Dlk1 *levels and Notch activity have been observed in cultured cells, it is possible that DLK1 could be a receptor antagonist [14].

The Notch pathway is highly conserved throughout the animal kingdom and has been most extensively studied in *Drosophila *where it was first identified [7,15]. Unlike mammals, which have 4 different Notch receptors, *Drosophila *has a single receptor and provides a simple system to test whether DLK1 has the capability to regulate Notch *in vivo *and whether it acts positively or negatively. This approach has successfully been used to investigate functions of bone fide mammalian Delta ligands, two of which were effective in activating *Drosophila *Notch [16]. We therefore generated transgenic flies that express *Dlk1 *isoforms under the control of the Gal4/UAS system [17] and tested for phenotypes indicative of alterations in Notch function.

## Results and Discussion

### Phenotypes from expressing Dlk1 in the Drosophila wing

Three different *Dlk1 *variants were constructed that mimic the different isoforms detected *in vivo*: S-Dlk, a secreted form produced by proteolytic cleavage in the extracellular domain, M-Dlk, transmembrane tethered form that lacks the cleavage site, SM-Dlk retains the cleavage site and thus has the capacity to be both membrane tethered and cleaved (Fig. 1). We used a series of different Gal4 driver lines to express these proteins in the developing wing and assessed the adults for phenotypes that would be indicative of effects on Notch activity.

Notch activity is required at many different stages in wing/notum development. Characteristic phenotypes of *Notch *gain of function are wing over-growth, ectopic wing margin structures, loss of veins and bristle loss [18-21]. Phenotypes caused by *Notch *loss of function are notching of the wing margin, vein thickening and bristle duplication/tufting or bristle loss [22,23]. The apparently contradictory effects on bristles occur because Notch is involved both in the selection of the sensory organ precursor (SOP) cells and in the fates within each bristle lineage [23]. Expression of *Drosophila *ligands in the wing produces characteristics of both Notch activation and inhibition. The latter occurs in the cells expressing highest levels of ligands, and is due to an autonomous inhibitory effect on the receptor referred to as cis-inhibition [9,24]. Expression of mammalian ligands in similar assays either resulted primarily in Notch activation (Dll1 and Dll4) or had no effect (Dll3), none had robust cis-inhibitory effects [16].

**Figure 2:**
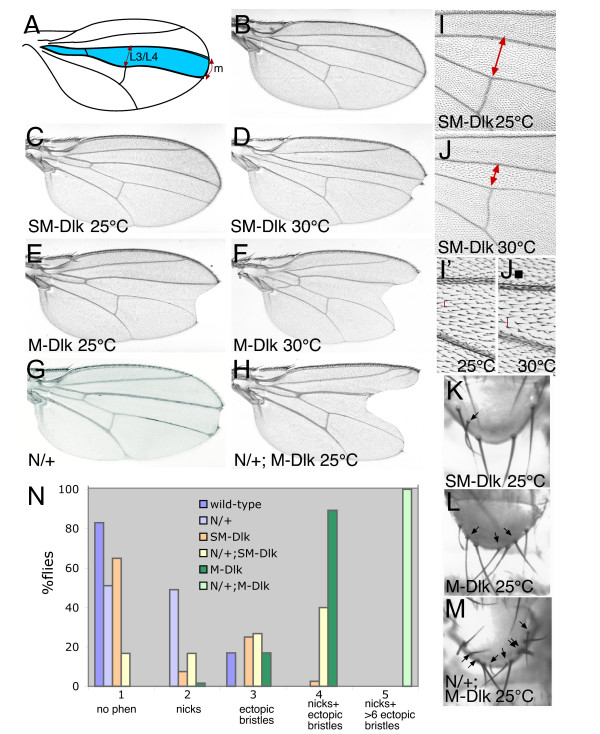
**Phenotypes caused by expression of Dlk1 resemble Notch loss-of-function and are enhanced by Notch mutations**. (A) Diagram showing domain of Ptc::Gal4 expression (blue) in the wing, red arrows indicate regions scored for Table 1 (m = margin). (B) Wild-type wing. (C-F) Wings expressing SM-Dlk (C,D; 2 copies of transgene) and M-Dlk (E,F; 1 copy of transgene). (G,H) Heterozygous *N*^55*e*11^/+ wings, phenotypes of M-Dlk expression (H) are enhanced. (I,J) Effects of SM-Dlk at 25°C (I,I') and 30°C (J,J') on cell number and cell size, cell number is reduced by stronger expression, (I,J; fewer cells between L3/L4, red arrows) and cell size is increased (I'J'; evident from spacing of the trichome hairs, e.g. red lines). (K-M) Ectopic sensory bristles (arrows, wild-type has 4 sensory bristles) in the scutellum of SM-expressing (K), and M-Dlk expressing flies (L,M). Ectopic bristles are enhanced when Notch levels are reduced (*N*^55*e*11^/+; M). (N) Graph summarising the phenotypes obtained with different combinations at 25°C. (SM-Dlk was present in 2 copies).

When DLK1 protein was expressed in the wing using several different Gal4 drivers we detected phenotypes that were consistent with a reduction in Notch activity, including wing notching, vein thickening and bristle duplications (Fig. 2 and data not shown). In all cases M-Dlk produced the strongest phenotypes, SM-Dlk resulted in much milder phenotypes and the secreted isoform, S-Dlk, had little or no effect. The latter may not be surprising because Notch ligands appear to require ubiquitinylation of the intracellular domain and coupling with endocytosis to be active [25-27]. The secreted isoform would not undergo the necessary modifications.

To further investigate the consequences of expressing *Dlk1 *we focussed on the *ptc::Gal4 *driver which is expressed in a stripe in the central region of the wing and in the scutellum (Fig. 2A,B). We used two approaches to vary the levels of expression. We tested effects at 25°C and 30°C, the higher temperature increasing the effectiveness of the Gal4, and we compared phenotypes from one and two copies of the transgenes. Expression from one copy of M-Dlk at 25°C resulted in highly penetrant phenotypes of wing notches and multiple sense-organs, indicative of reduced Notch activity (Fig. 2E,F,M,N). Both defects were strongly enhanced at 30°C. In contrast, minor defects of occasional bristle duplications were produced using one copy of SM-Dlk (data not shown). The bristle defects were more penetrant with two copies of SM-Dlk and occasional wings had mild-notching (Fig. 2N). These effects were considerably enhanced at 30°C (Fig. 1D,N). Thus both M-Dlk and SM-Dlk produce defects consistent with an antagonistic effect on Notch. The transgene producing the membrane tethered M-Dlk has a much more potent effect.

In addition to the Notch-related defects, we also observed effects on cell proliferation. In particular, expression of SM-Dlk resulted in fewer cells in the domain of expression (Fig. 2I,J and Table 1). Within the reduced domain the individual cells were larger than normal. In contrast, expression of the M-Dlk caused a slight increase in cell number in the domain of expression (Fig 2F,N). Together these data suggest that DLK1 affects cell growth or proliferation, and that the different isoforms may have distinct inputs on this process. Furthermore, although there is evidence that Notch can influence cell proliferation in the wing [28], effects on cell size were not seen in other experiments where Notch activity was modulated by *ptc::*Gal4 (e.g. with dominant negative ligands or with Su(H)). Thus, effects on cell size seen with SM-Dlk may be suggestive of additional target(s) or may reveal a novel aspect of Notch function.

**Table 1:** Cell numbers (+/- s.d.) in two regions of wings from wild type flies and flies expressing Dlk isoforms.

	**Wild type**	**SM 30°C**	**SM 25°C**	***N*/+; SM 30°C**	***N*/+; SM 25°C**	**M 25°C**
**Margin**	32.44(+/- 1.59)	22.41(+/- 2.92)	31.58(+/- 2.09)	Nicks	Nicks	Nicks
**L3/L4**	15.22(+/- .83)	10.13(+/- 1.09)	14.86(+/- 0.77)	11.88(+/- 0.88)	14.75(+/- 0.89	17.08(+/- 1.31)

N ≥ 12 flies per genotype. See Figure 2 for areas used for counting. Reducing the dosage of *Notch *(columns labelled *N/+*) does not influence cell numbers.

### Some Dlk1 expression phenotypes are enhanced by Notch mutations

To confirm that phenotypes produced by M-Dlk and SM-Dlk are due to effects on Notch, we asked whether they could be enhanced if the levels of Notch were reduced in flies heterozygous for a loss-of-function *Notch *allele (*N*^55*e*11^). Heterozygosity for *Notch *alone produces mild wing nicking, slight broadening of the veins at the tips (deltas) and occasional duplication of the scutellar macrochaetae (Fig. 2G and data not shown). Even taking into account the effects of Notch mutation on the wing margin, the frequency and the extent of wing notching from both M-Dlk and SM-Dlk was enhanced in N/+ females (Fig. 2H,N). Furthermore, the effects of bristle duplication were also significantly enhanced (Fig. 2I–N). Thus by two different criteria, the phenotypes produced by DLK1 are enhanced by reducing the amount of Notch protein present, indicating that DLK1 is antagonising the activity of the Drosophila Notch receptor.

Although most of the effects of *Dlk1 *expression are enhanced by *Notch *mutations, the wings from *Notch*/+; *ptc::Gal4*/*UAS::SM-Dlk *have a similar size and number of cells in the domain of expression to those with wild-type dose of *Notch*. This is consistent with the hypothesis that the effects on cell size are independent of an action on Notch raising the possibility that there is an additional target of DLK1.

### Effects of Dlk1 expression on Notch targets in the wing imaginal disk

**Figure 3:**
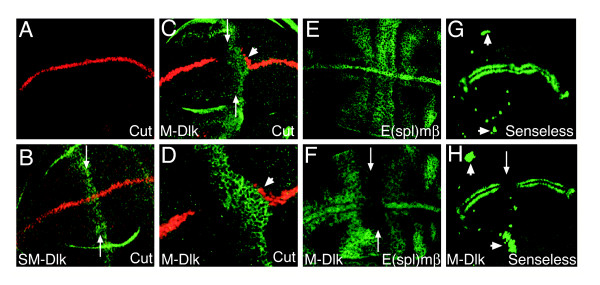
**Expression of Dlk1 inhibits Notch target genes and leads to ectopic sensory organ precursors**. (A) Wild-type wing imaginal disc, Cut (red) is detected in a stripe along the d/v boundary. (B) Expression of SM-Dlk (green, arrows) leads to a slight reduction in Cut (red). (C,D) Expression of M-Dlk (green, arrows) inhibits Cut (red) expression. *ptc::Gal4 *is expressed in a slight gradient, barely detectable levels of DLK1 in the anterior of the domain are still sufficient to inhibit Cut. A few cells where Cut is induced ectopically are detected posterior to the DLK1 expression domain (arrowhead). D is a higher magnification of C, note that the M-Dlk is seen at the membrane of the expressing cells. (E,F) *E(spl)mβ::CD2 *expression in wild-type (E) and M-Dlk expressing disc. Arrows in F indicate the domain of M-Dlk expression where *E(spl)mβ::CD2 *is inhibited. (G,H) Senseless expression in wild-type (G) and M-Dlk expressing disc. Senseless expression is inhibited at the d/v boundary (arrows; characteristic of loss of Notch activity) and is expanded in the ventral and dorsal radius (arrowheads) indicative of ectopic sensory organ precursors.

If DLK1 is antagonising Notch activity we would expect to see down-regulation of Notch target genes in the wing imaginal disc. We examined effects of expressing M-Dlk and SM-Dlk on two Notch targets. Expression of the transcription factor Cut at the wing margin (d/v boundary; Fig. 3A) is dependent on the activity of the Notch and Wingless pathways [29]. The *cut *regulatory sequences contain binding sites for the DNA-binding protein Su(H) (the intracellular transducer of Notch activity, homologous to CBF1 in mammals) suggesting that it is a direct target of Notch activation [30]. Cut expression was strongly inhibited by expression of M-Dlk and subtly reduced by expression of SM-Dlk (Fig. 3B–D). In addition, occasionally there was some ectopic Cut detected at the boundary of M-Dlk expression in the ventral part of the wing (Fig. 3C,D). This suggests that M-Dlk may also have some activating potential but only in the ventral domain, where the Fringe glycosyl-transferase is absent. In this respect Dlk-1 appears more similar in function to Serrate, which is only able to activate Notch molecules that are not modified by Fringe [31].

The second target used was the *Enhancer of split mβ *gene (*E(spl)mβ*), which is expressed in response to Notch in a more widespread pattern in the wing (Fig. 3E; [32,33]. We have previously mapped the regulatory sequences of this gene and shown that its expression is dependent on Notch activity [32]. Using a reporter construct where the regulatory sequences drive expression of a heterologous protein, the membrane protein CD2 [34] we tested effects of *Dlk1 *expression. M-Dlk strongly down-regulated *E(spl)mβ*::CD2 throughout the domain of misexpression (Fig. 3F; occasionally this was accompanied by slight upregulation at the boundary) SM-Dlk had weaker but still significant inhibitory effects (data not shown). The inhibition of both *E(spl)mβ *and *cut *expression confirms that expression of *Dlk1 *is antagonising Notch activity. As this inhibition occurs within the domain of ligand expression, it most likely represents cis-inhibition, i.e. effects on the receptor in the same cell. However we cannot rule out the possibility that there are slight non-autonomous inhibitory effects with M-Dlk, as the epithelium often becomes deformed around the domain of expression.

We also wanted to verify that the effects of *Dlk1 *on bristles reflect a reduction in Notch activity, because some of the adult phenotypes (most notably bristle loss) can also be brought about by increased Notch activity. We therefore looked at a marker of sensory-organ precursors, Senseless [35]. If DLK1 is antagonising Notch activity we would expect an increase the number of sensory organ precursors in a given cluster. This is what was observed in the clusters that will form the chordotonal sensory organs of the dorsal and ventral radius (Fig. 3G,H). Both M-Dlk and SM-Dlk increase the number of Senseless-expressing precursors in these clusters, with M-Dlk having the stronger effect (Fig 3H and data not shown).

### M-Dlk and SM-Dlk show different cellular locations and effects on Notch protein

**Figure 4:**
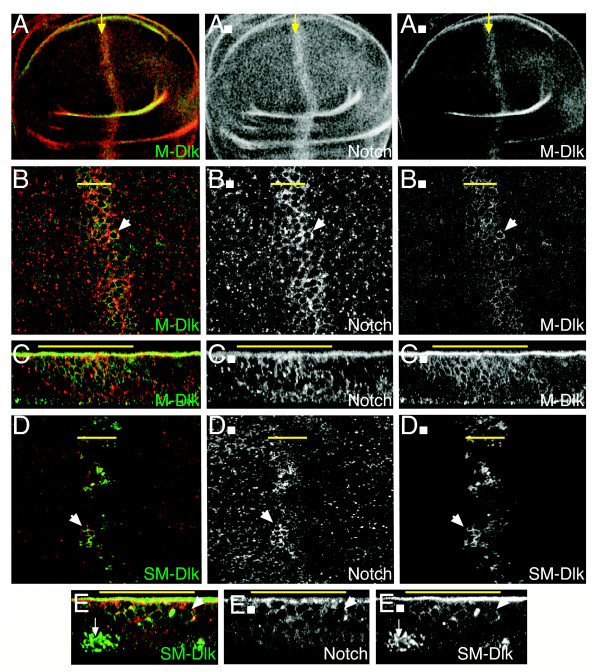
**Expression of Dlk1 alters the cellular distribution of Notch**. (A-C) M-Dlk (A,A" green, white) is present at the cortex/membrane of expressing cells and results in stabilization of Notch (A,A', B,B', C,C' red, white) within the stripe of M-Dlk expression (yellow arrows/line). (B-B") Higher magnification, cell outlines with M-Dlk and Notch enrichment are visible (e.g. arrowhead). (C-C") X/Z section, co-enrichment of Notch and M-Dlk on apical and lateral regions of cells is seen (e.g. arrowhead; note there is also non-specific accumulation of anti-Dlk staining along the surface of the specimen). (D-E) SM-Dlk (D, D"; E, E"; green, white) accumulates on the basal surface of the epithelium (e.g. arrow in E,E"), and at lower levels around the membrane/cortical regions. Less stabilisation/accumulation of Notch is detected, but there is enrichment at the cortex of some SM-Dlk expressing cells (e.g. arrowheads). (A'-E') Notch channels only, (A"-E") anti-Dlk channels only. Yellow arrows and lines indicate domain of *ptc::Gal4 *driven expression.

The differing severity of the M-Dlk and SM-Dlk might in part be due to the transgenes being expressed at different levels. However, the fact that even increasing the dosage and temperature failed to properly redress the difference, and that the two proteins had distinct effects on proliferation suggested that there are intrinsic differences in the behaviours of the proteins. To investigate these questions we stained wing discs with an antibody that recognises DLK1. This revealed a striking difference in the behaviour of the two proteins. M-Dlk had a cortical/membrane distribution (Fig. 4B,B',C,C'), whereas SM-Dlk accumulated at highest levels basally, although some membrane associated protein was detectable (Fig. 4D,D",E,E").

Co-staining with anti-Notch antibody revealed that M-Dlk strongly affects the distribution of the endogenous Notch protein (Fig. 4A',B',C'). The levels of Notch are elevated in M-Dlk expressing cells, and there is more protein accumulating at or close to the membrane of these cells. Both these effects suggest that M-Dlk has an action on Notch that stabilises the protein. Notch distribution is much less affected by SM-Dlk, despite the fact that this isoform consistently accumulates to much higher levels than M-Dlk (Fig. 4D'). Nevertheless in some cells we also observed redistribution of Notch to co-localise with the SM-Dlk (Fig. 4D',E'). We take this co-variance in Notch localisation with the expressed Dlk isoforms to indicate that they are able to interact, since this resembles the effects seen with expression of known ligands with functional DSL domains [9]. In contrast, in these previous studies, ligands with a mutated DSL domain were not able to influence Notch distribution [9].

## Conclusion

### Dlk1 can regulate Notch signalling despite its atypical structure

Our data indicate that DLK1 has the capability to regulate Notch receptors in spite of the fact that it lacks an N-terminal DSL domain. This *in vivo *functional demonstration that DLK1 can affect *Drosophila *Notch signalling and is consistent with yeast two-hybrid data showing interaction of DLK1 with Notch1 EGF repeats [10]. That this interaction has likely functional consequences in mammals *in vivo*, is supported by evidence suggesting that DLK1 can modulate levels of Notch signalling in adipogenic cells in culture [14]. In our assay the effects of DLK1 appear to be largely inhibitory. This is in contrast to the effects seen when the typical mammalian Delta ligands *Dll1*, *Dll3 *and *Dll4 *were expressed in flies [16]. In those experiments, both DLL1 and DLL4 were able to activate Drosophila Notch, and DLL3 had no effect. Neither DLL3 not the other two ligands produced inhibitory effects similar to those seen here with DLK1. Thus, these effects are likely to reflect specific characteristics of DLK1 itself.

Notch ligands have the unusual characteristic that they inhibit the Notch receptors present on the same cell, as well as activating receptors on adjacent cells [24,36]. It is the autonomous cis-inhibitory activity that we detect with DLK1, with little or no evidence for activation. Furthermore, the effects are most profound when we use a membrane-tethered form of DLK1 (M-Dlk). However, our results with DLK1 do not *a priori *indicate that DLK1 acts solely as an inhibitor at the membrane. The ability of ligands to activate the Notch receptors is affected by glycosylation [37] and it is possible that the glycosylation state of the Drosophila Notch is incompatible with DLK1 activation. It is also possible that species-specific differences in endocytosis, in interactions with adaptor proteins, or in ligand specificities might also influence the outcome in our experiments. Furthermore, mammalian cells have 4 Notch receptors, and while two of these (Notch 1 and Notch 2) contain the same number of extracellular EGF repeats as Drosophila Notch, the others (Notch 3 and 4) have fewer and may have different interactions/ligand pairings. Thus the outcome of DLK1 actions could differ according to which specific Notch receptor or ligand-receptor pairing is affected. Nevertheless our results do show that DLK1 has the ability to regulate Notch signalling particularly when membrane tethered; hence it is likely to be an important factor acting on this pathway in mammalian development.

During mammalian embryogenesis, *Dlk1 *is expressed in many of the key lineages known to depend on Notch signalling for appropriate development, including somite derivatives [38]. The relative expression of *Dlk1 *and the more typical Notch ligands may therefore play an important role in modulating the temporal and spatial control of Notch signalling in these cells. Once the expression and function of DLK1 *in situ *is better characterised, this may shed light on which of the mammalian receptors is likely to be a target of DLK1, what the functional relationships between DLK1 and other Notch ligands are, and whether DLK1 acts positively as well as antagonistically on Notch receptors.

## Methods

### Dlk1 constructs and transgenic flies

Three constructs were generated, representing the predominant forms of *Dlk1 *that are generated by alternate splicing and proteolysis. Genomic fragments were generated from BAC103N10 (Accession number AJ320506) and combined with appropriate fragments from a full length cDNA clone containing the *Dlk1 *open reading frame (IMAGE 604466) but lacking the protease cleavage domain. Open reading frames were ligated into pUAST for injection into *yellow white *flies. The first construct contained the largest open reading frame harbouring the intracellular and proteolytic cleavage domains (SM-Dlk), the second lacked the cleavage domain (M-Dlk), the third contained only the extracellular domain, equivalent to the secreted form of DLK1 after cleavage (S-Dlk). This was generated from the M form by truncating 5' of the proteolytic cleavage domain (via Sac I digestion), prior to cloning into pUAST. All constructs were sequenced to confirm accurate synthesis. Several independent lines were isolated and 3 lines of each were selected for further analysis, based on expression levels (antibody staining) and strength of phenotypes. All lines for a given construct gave qualitatively similar results. Homozygous stocks were prepared containing insertions on chromosomes II and III and these were combined for experiments with 2 copies of the transgenes.

### Fly stocks and analysis of Adult phenotypes

Except where otherwise stated fly stocks used are as described [39]. Adult flies were stored in 100% ethanol and selected parts were mounted in Euparol for photography.

### Immunofluorescence

Dissection of larvae and immunofluorescence were as described previously [40] except that dissections were carried out in PBS. Primary antibodies were rabbit anti-DLK1 [41]; mouse anti-Senseless [35]; mouse anti-Cut (1/20; [42]); mouse anti-Notch (C17.9C6 1/20; [43]. The latter two antibodies were obtained from Developmental Studies Hybridoma Bank. Secondary antibodies were from Jackson Immnunological. Images were collected using a Leica TCS-NT-UV scanning confocal microscope.

## Authors' contributions

SJB and AFS designed the experiments. SJB, ST, EH and S-CS carried out the experiments, SJB and AFS analysed the data, and wrote the manuscript. All authors read and approved the final manuscript.
